# Optimizing the GATA-3 position weight matrix to improve the identification of novel binding sites

**DOI:** 10.1186/1471-2164-13-416

**Published:** 2012-08-22

**Authors:** Soumyadeep Nandi, Ilya Ioshikhes

**Affiliations:** 1 Ottawa Institute of Systems Biology and Department of Biochemistry, Microbiology and Immunology, Faculty of Medicine, University of Ottawa, Ottawa, Ontario, Canada

**Keywords:** Transcription factor, Binding sites, GATA-3, Human promoter, Position weight matrix, Optimization

## Abstract

**Background:**

The identifying of binding sites for transcription factors is a key component of gene regulatory network analysis. This is often done using position-weight matrices (PWMs). Because of the importance of *in silico* mapping of tentative binding sites, we previously developed an approach for PWM optimization that substantially improves the accuracy of such mapping.

**Results:**

The present work implements the optimization algorithm applied to the existing PWM for GATA-3 transcription factor and builds a new di-nucleotide PWM. The existing available PWM is based on experimental data adopted from Jaspar. The optimized PWM substantially improves the sensitivity and specificity of the TF mapping compared to the conventional applications. The refined PWM also facilitates *in silico* identification of novel binding sites that are supported by experimental data. We also describe uncommon positioning of binding motifs for several T-cell lineage specific factors in human promoters.

**Conclusion:**

Our proposed di-nucleotide PWM approach outperforms the conventional mono-nucleotide PWM approach with respect to GATA-3. Therefore our new di-nucleotide PWM provides new insight into plausible transcriptional regulatory interactions in human promoters.

## Background

Understanding the regulation of gene expression is a complex problem and one of the most challenging domains of biological and biomedical research. Intensive ongoing studies aim to understand the detailed mechanisms of the transcriptional regulation in eukaryotes. Transcription factors (TFs) are proteins that regulate the activity of a gene at the levels of mRNA synthesis. These factors bind to specific DNA sequences at positions in the genome near the gene and either reduce or enhance its transcription rate [1]. The binding of TFs to DNA requires specific short cis-regulatory sequences (binding sites), usually located upstream of 5' end of the gene (gene promoter). Binding sites may also be located in the promoter proximal region, or more distally from the target gene [2]. Different DNA binding sites for a specific TF often share common features called sequence motifs [3]. The binding site motifs are often highly degenerate, which makes it challenging to build reliable models for these DNA-encoded signals [4]. A common approach to build these models is use of position weight matrices (PWMs) [5-13].

A crucial limitation of the PWM approach is the paucity of a sufficient number of high confidence, experimentally verified binding sites. One way to address this problem is to include additional transcription factor binding sites (TFBS) identified computationally by including genomic sequences with substantial similarity to the PWM of a particular TF [14,15].

Several methods to build PWMs have been described. One of the most successful methods was proposed by Staden [8]. It uses a collection of aligned TFBS to calculate a base frequency table. The table comprises four rows for each nucleotide (A, T, G and C) and the columns represent the length of the binding sites. The weight matrix represents the logarithms of the probabilities of finding each base at each position in a signal. Correspondingly, the PWM is the estimate of the log-probabilities of each base occurring at each position in the aligned TFBS.

The Staden method does not include the definition of the optimal cutoff to minimize a level of false positive predictions for a given level of true positives [4]. Bucher described a method to optimize the cutoff value of the PWM [16] that was extended by Tsunoda and Takagi [17]. They calculated the optimal cutoff values for 205 vertebrate TFs from TRANSFAC. The method proposed by Gershenzon et al. [18] is another extension of the Staden-Bucher method [8,16]. It optimizes various PWM parameters including the cutoff and calculates sensitivity and specificity of the derived PWM [19,20]. In the present study we adopt the method by Gershenzon et al. [18] first to use PWM built on experimental binding site data from Jaspar to identify probable GATA-3 binding sites within promoters, and subsequently to incorporate additional binding site information into the PWM, hereby achieving better sensitivity and/or specificity of the putative binding motif prediction by the optimized PWM. Analysis of high throughput ChIP data opens additional opportunities of PWM optimization. The study by Leping et al. [21] considered the ChIP data (human and mouse Oct4 and human p53) for PWM optimization using genetic algorithm. However, the main problem of using ChIP data for PWM optimization is its low resolution which may result in high level of false positive predictions by the optimized PWM. To overcome this problem, we consider the GATA-3 binding sites as more likely to be located in the relatively narrow area of a promoter region. Our method would be also useful for optimization of TFs whose ChIP data is not yet available.

A standard PWM approach is based on the assumption that individual nucleotides contribute independently and additively to the binding of a TF to a given DNA motif [3]. Yet previous studies [18,22-24] demonstrate that some TFBS nucleotides are mutually dependent. To account for such non-additive effects we proposed that di-nucleotide PWMs may be more accurate [18]. In our analysis, we optimize both the mono-nucleotide and the di-nucleotide matrices. (See Materials and Methods for details.)

We implemented the Gershenzon's method [18] to analyze the known binding sites for GATA-3 and to identify novel GATA-3 TFBS. We selected this TF because of its important role in the T-cell development [25,26] and the differentiation of T-cells into effector subset [27]. The factor is involved in three differentiation steps: specification, T cell receptor (TCRαβ)-dependent positive selection, and the activation of T helper cell (Th2) programs in mature T-cells. In addition, the method we adopted [18] originally dealt with Sp1 factor which has a broad positional distribution of binding motifs with a single peak around TSS in the interval (-499 to +100 bp). However, GATA-3 occurrence distribution has two peaks instead of one. We compared the distributions of several factors and found that they exhibit either one peak in the promoter area like ubiquitous Sp1 and E2F or two peaks like GATA-3, TCF1 and Ets-1 specific for T-cell lineage. Hence GATA-3 is an attractive candidate for this study, also because it may be considered as a typical representative of variety of T-cell specific TFs with specific positional distribution.

From the binding sites discovered in the present study some were previously confirmed to be the important binding sites [28-30] for GATA-3, as mutating them causes complete loss of enhancer activity [30]. Nonetheless, they are not incorporated in existing databases of TFBSs and thus were neither a part of the original PWM nor were predicted by it. Identification of these sites by our optimized PWM provides experimental evidence of superiority of our TFBS prediction approach versus existing techniques.

## Results

### GATA-3 overrepresentation in the promoter area

**Figure 1:**
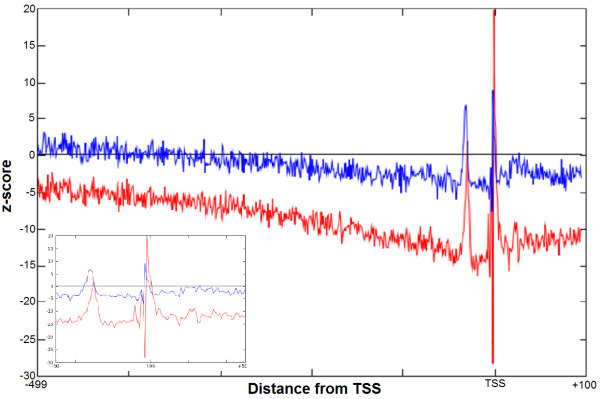
**Distribution of the occurrence frequency of GATA-3 in two different human promoter databases.** Occurrence frequency of the GATA-3 motifs in the promoter databases. x-axis represents distance (bp) with regards to TSS and y-axis represents the z-score. Red line denotes the z-score distribution of OF in EPD and blue is for DBTSS. The promoters were aligned with respect to the TSS which is at 0. The horizontal line denotes the OF_r_ in the shuffled promoter database which is derived from EPD promoter database. The inset figure shows the distribution area from -50 bp to +50 bp to clearly reveal both the peaks.

We started with computational identification of the GATA-3 binding motifs in the promoters, and then we determined the region where the GATA-3 sites are statistically over-represented. Following [18] we term this region as a functional interval (window) for GATA-3 deeming the statistical over-representation as functionally related. First we examined the spatial distribution of the occurrence frequencies (OF) of the GATA-3 motif in two promoter databases: EPD [31-33] with 1870 human promoter sequences and DBTSS [34,35] with 32102 promoter sequences. Promoter sequences in both databases were aligned with respect to the TSS. We scanned these promoter sequence databases with the original PWM for GATA-3. We quantified the distributions in terms of z-scores, which quantitate the difference between the occurrence frequency distribution of GATA-3 motif in actual promoter sequences (OF) and the shuffled background sequences (OF_r_ see Methods, Formula 6). Since positional distribution of GATA-3 was basically consistent for both databases (Figure 1), we further mostly focused on the analysis of the EPD, because this database is being maintained as strictly non-redundant database with its promoters rigorously selected, curated and quality-controlled. However, analysis of the DBTSS was also performed for verification purposes with results basically consistent with those by the EPD. From Figure 1 we can see that the z-score distribution for EPD (blue) and DBTSS (red) have quite similar features. Since the z-score is proportional to the square root of number of sequences in the promoter database (Formula 6), the z-score may increase with higher number of sequences in DBTSS. This explains why the EPD curve is below the DBTSS curve. The distributions are under-represented almost across the entire promoter region with few exceptions in the immediate upstream areas. We can see two peaks: one around -2 bp (-1 bp to -3 bp) upstream of the TSS at 0 and another further upstream at -31 bp (-29 bp to -33 bp). (The positions represent the beginning of the motif.) The peaks are present in both the curves, for EPD and DBTSS, and coincide in the same area in both the distributions. In our study we utilized the area 500 bp upstream of TSS as no peaks are seen upstream of the -500 bp region up to -10 kb interval ( Additional file 1: Figure S1).

**Figure 2:**
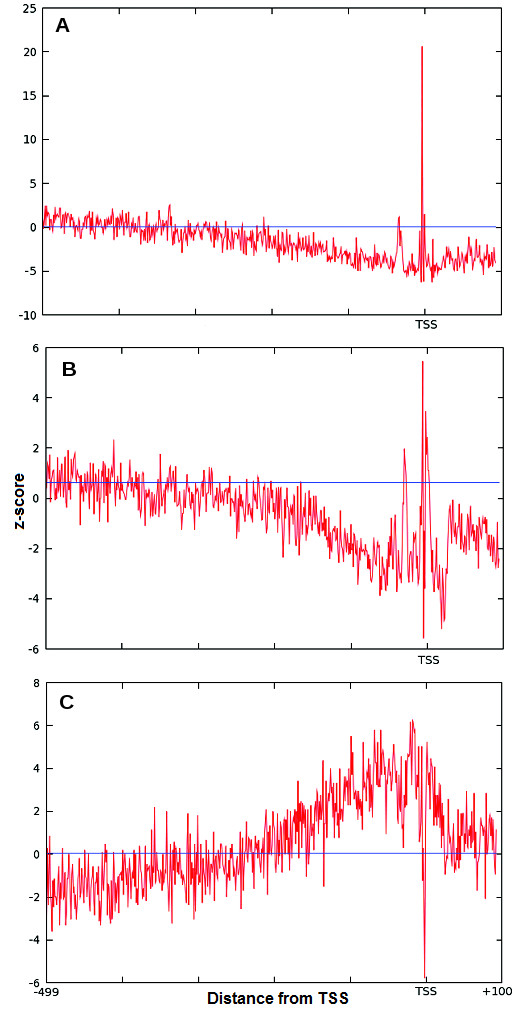
**Distribution of the occurrence frequency of TCF-1, Ets-1 and E2F in human promoter database (EPD) in the proximal promoter region.** The individual occurrence frequency distributions of the various transcription factors in human promoters from EPD. The y-axis is the OF of the transcription factors TCF-1 (**A**), Ets-1 (**B**) and E2F (**C**) motifs. The x-axis shows the areas upstream and downstream of the TSS which is at 0. The blue horizontal line shows the expected occurrence frequency OF_r_ for each of the factors calculated from the shuffled sequence dataset.

**Figure 3:**
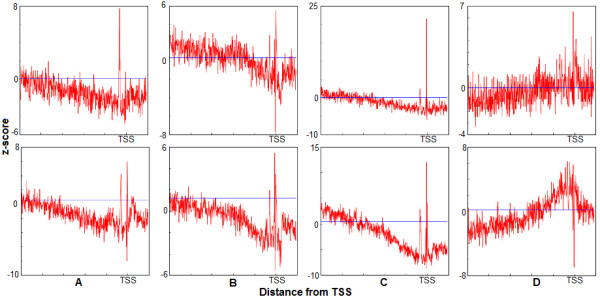
**Differences in the distribution of the occurrence frequency of transcription factors at different thresholds (relaxed and strict).** The comparison of occurrence frequencies of the four transcription factors (GATA-3, Ets-1, TCF-1 and E2F) for different thresholds. The axes are same as at the Figure 1. The top row is the z-score distribution at a strict threshold. The second row is the z-score distribution with relaxed threshold. In all these plots we can see two distinct peaks and the depression area around the peaks. The straight blue horizontal line shows the expected occurrence frequency OF_r_ from the shuffled dataset.

The difference between the occurrence frequency of the GATA-3 motif in the promoter sequences and occurrence frequency in the shuffled promoter database (shown in the plot as the horizontal line) is much higher in the peak areas than in the rest of the promoter interval. The plot prominently identifies the over-represented area in the promoter with z-score of ~3. The GATA-3 sites are functional in either orientation [30,36]. Therefore the GATA-3 motif was scanned using the original PWM in both strands. Since these over-representation windows are located in the proximal upstream region, they may have functional importance for GATA-3. Therefore, GATA-3 TFBS motifs located inside these windows may be utilized to improve the PWM for GATA-3. The previous work mentioned the presence of single over-represented area for Sp1 transcription factor in the proximal region of the human promoters [18]. The occurrence of two peaks for GATA-3 is somewhat unexpected. To verify the presence of the double peak in the distribution, other similar transcription factors were also examined. GATA-3 is a T-cell/kidney/brain lineage specific factor [37], so to verify this distribution pattern for other T-cell lineage specific factors we have studied the distribution of Ets-1 and TCF-1 [37]. As we can see from the Figure 2A and 2B, these two transcription factors also have similar positional distributions. They too have double peaks, one around -32 and another just upstream of the TSS. However, the peak around -32 is always smaller than those downstream. Importantly both the plots show the distinct two peaks. To compare with a transcription factor that is not T-cell specific we have selected E2F. The z-score distribution of this factor does not exhibit double peaks. In addition to the peaks, we have observed one more phenomenon in the distribution of these transcription factors. If we compare two z-score distributions of the same transcription factor, one with stringent cutoff and another with relaxed cutoff, the expected frequencies in these z-score distributions are raised to higher values for the latter, which is expected, as more false positive motifs would be picked up with a lower threshold (Figure 3). However, the immediate surrounding area of the peak remains under-represented with the relaxed threshold for GATA-3, Ets-1 and TCF-1. For TCF-1 the under-representation of the motif in the bottom panel around the peak area is more prominent compared with the upper panel. A possible explanation of this phenomenon is that the sequences around the binding sites make the region unfavorable for the transcription factor to bind in this area, which on the other hand may enable the transcription factor to locate its exact binding site.

As seen from the z-score distribution (Figure 1), there is a slope from the further upstream of -499 bp up to the area around the functional window. The slope becomes more prominent if the threshold is relaxed. The slope may be the result of functional binding sites present more upstream of the promoter which we might have missed in the length of the promoter region being studied (-499 to +100). To confirm that there is no other functional window present further upstream of the considered promoter region in this study, we have checked the distribution of z-score up to 10 kb upstream. In such distant area we could not see any peak apart from the peak just upstream of the TSS ( Additional file 1: Figure S1).

**Figure 4:**
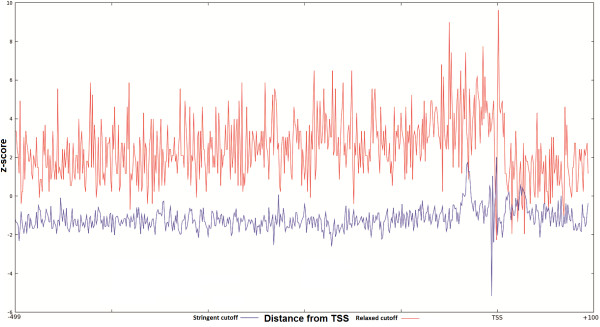
**z-score distribution for Pu1 transcription factor across the promoter sequences from EPD (-499 to +100).** Blue plot is the distribution of z-score with relaxed threshold and red is with stringent threshold. X-axis represents distance with regards to TSS(bp) and y-axis represents the z-score.

**Figure 5:**
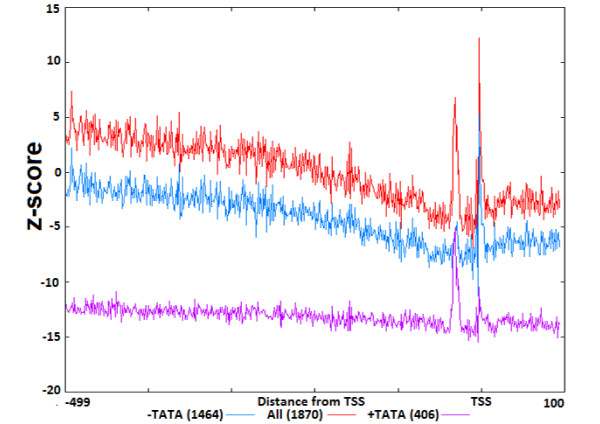
**Comparison of the GATA-3 z-score distribution in promoters divided with respect to the presence of TATA-box.** Distribution of the GATA-3 motif in partitioned EPD promoters based on the presence and absence of TATA-box element. Magenta and blue line represent the distribution of GATA-3 in promoters containing (406) and lacking (1464) TATA-box respectively and red line represents the distribution in all promoters (1870). X-axis represents distance with regards to TSS (bp) and y-axis represents the z-score.

Furthermore, to investigate the reason of the slope we also checked the z-score distribution of other transcription factors, namely Sp1 and Pu1 (Figure 4). In these plots the z-score distributions do not show the same behavior as for GATA-3, Ets-1 and TCF-1 i.e. there is no slope or under-represented area around the over-represented region of binding sites. This visible slope can be explained by the fact that 72% of the promoters in human genome are CpG rich regions [38]. This can further be confirmed from the binding sites distribution of GC-rich transcription factors like E2F in Figure 3 and Sp1 and Pu1 in Figure 4. The slope is the representation of the difference between the GATA-3 distribution in the actual promoters and in the shuffled sequences. To correctly obtain the z-score distribution we took into account the relatively rich concentration of CpG di-nucleotides in the promoters of human and generated our shuffled dataset by preserving the proportion same. From the z-score distribution we can see two peaks: one immediately upstream or over the TSS and another further upstream around position -31. To verify if the latter peak is caused by the presence of a similar TATA motif we have classified the promoters into two groups: TATA containing (TATA+) and TATA-less (TATA-) group using program Promoter Classifier [39]. In both the groups we have analyzed the z-score distribution of the predicted GATA-3 motif. In TATA containing promoters the peak in position -31 is substantially higher than the peak over TSS. This implies that the peak around -31 is mainly due to the TATA-like motif enrichment. Similarly in TATA-less promoters the peak over TSS is substantially higher than the peak at -31 (Figure 5). Thus the peak in the area around the TSS is most likely related to the GATA-3 binding.

**Figure 6:**
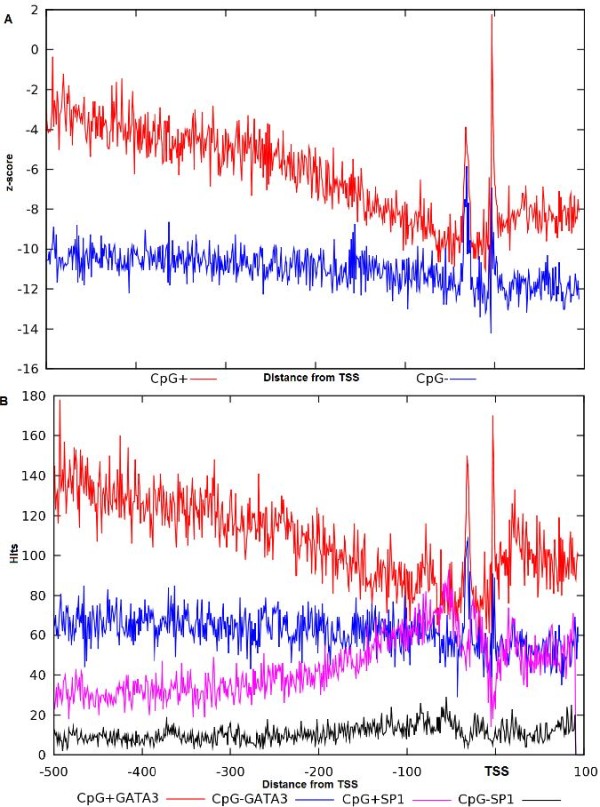
**Comparison of the GATA-3 z-score distribution in promoters divided with respect to the presence of CpG islands.** Distribution of the GATA-3 and Sp1 motif across the EPD promoters divided based on the presence and absence of CpG-island. **A**) Comparison of the distribution of GATA-3 in promoters containing CpG-island (red plot) and promoters lacking CpG-island (blue plot). X-axis represents distance (bp) with regards to TSS and y-axis represents the z-score. **B**) Comparison of the distribution of hits of GATA-3 and Sp1. GATA-3 hits are represented with red and blue in CpG-island present and absent promoters respectively and magenta and black represent the distribution in CpG-island present and absent promoters respectively. X-axis represents distance (bp) with regards to TSS and y-axis represents the hits.

However, we have also classified the promoters to the CpG-island-containing (CpG+) and non-CpG-island-containing (CpG-) groups with the same method [39]. From this analysis it is clear that the slope that we encountered in the promoter region is due to the CpG rich area (Figure 6A). The GATA-3 motifs are found in abundance in CpG rich promoters as one can see from the graph. The peak around -2 bp from TSS of the z-score distribution of GATA-3 in CpG+ promoters (red plot) is much higher than those in the CpG- promoters (blue plot). In addition we have also analyzed the distribution of hits in the two datasets (CpG+ and CpG-). The distributions of hits in these two datasets are similar to that of the z-score distribution. In the CpG+ promoters the slope is more prominent than in the CpG- promoters. To verify whether the slope is caused by the GC rich content of the human promoters, we have analyzed the distribution of hits for GC rich factor. For this purpose, we have compared the distribution of GC rich factor Sp1 with GATA-3 distribution. Indeed the Sp1 factor’s distribution is just the opposite to that of the GATA-3. This can be seen in the Figure 6B, where the hits distributions are plotted for both GATA-3 and Sp1. The region where GATA-3 is under-represented is over-represented with Sp1 hits and vice versa.

This means that the slope in the plot is caused by the presence of CpG-islands in the promoters. The over-representation of GATA-3 motif over TSS is caused by the genuine GATA-3-like motif rich area whereas the peak further upstream around -31 bp from TSS is caused by the TATA box rich area.

### New PWM for the GATA-3

To start the optimization we built a PWM from the binding sites from Jaspar [40]. Since the cutoff for the existing matrix is not given, we determined the initial cutoff as described in the Methods section. Providing the original matrix with the determined cutoff value, the set of experimentally defined GATA-3 binding sites and initial functional window to the optimization procedure, we attained a new matrix. We performed the optimization for both aforementioned intervals (-3 to -1) and (-33 to -29) separately. During the process of optimization from the former interval (-3 to -1) the process attained the new window (-7 to 0) including the transcription start site at 0 where putative binding sites are statistically over-represented (with z > 3) in promoter sequences versus control dataset and from where new binding sites were selected to build the new PWM. This interval represents optimized “functional window” for GATA-3 as defined above. (The same process of optimization with the interval around (-33 to -29) resulted in a lower sensitivity and specificity for the optimized PWM.)

Both the mono-nucleotide matrix and the di-nucleotide matrix were optimized in the same window. The determined initial cutoff value for the original mono-nucleotide matrix is -1.0 with the sensitivity 50%. The two new matrices were optimized with two different cutoff values: -2.0 for the mono-nucleotide and -3.5 for the di-nucleotide matrix. We compared the performance of the initial PWM built from the Jaspar binding sites for GATA-3 as described in the Methods section, as well as those for the new mono-nucleotide and di-nucleotide PWMs with different levels of sensitivity with the performance of Match program (TFBS search algorithm from TRANSFAC) for accession id M00077. Receiver-Operator Characteristic (ROC) curves which plot the true positive rate vs. false positive rate (specificity vs. sensitivity) are usually used to compare different classifiers [41]. They use numbers of true positive, false negative and false positive predictions. Since we don’t have actual number of the false positives, we use the parameter of Occurrence Frequency (OF_r_) of predicted sites picked from the shuffled sequences as a level of false positives (Formula 8).

**Figure 7:**
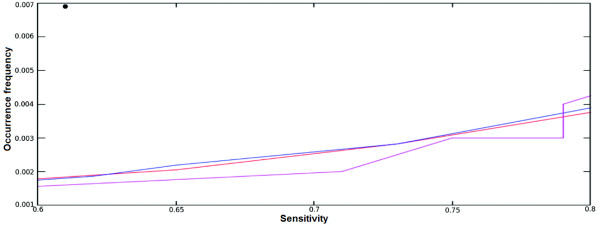
**Comparison of PWM (mono-nucleotide and di-nucleotide) with the program Match.** The OF_r_ and sensitivity comparison for different PWM and Match. Occurrence frequencies are at Y-axis. X-axis denotes the sensitivities. The average OF_r_ of the Match tool is denoted as filled circle (upper left corner). The sensitivity and specificity curve for the PWM obtained from GATA-3 binding sites from Jaspar is represented by blue, red and magenta are of the new mono-nucleotide PWM and di-nucleotide PWM respectively.

Figure 7 represents the OF_r_ versus sensitivity (percentage of sites selected from the experimentally verified motifs). The Match program uses three matrix-specific cutoffs which attempt to minimize either false-negative error (minFN), false-positive error (minFP), or the sum of these two errors (minSUM) [42]. If we compare the Match results (dot at the left upper corner) with those by our original matrix (blue line), new mono-nucleotide (red line) and di-nucleotide (magenta) matrices, we find that the randomized OF_r_ for Match is much higher comparing to the other PWMs for the sensitivity around 60%. Here, the Match program was run with the minimum FN cutoff provided in the TRANSFAC. The sensitivity of the Match for other thresholds was very low (~15% and ~30% for minimal FP and SUM, respectively). The original PWM obtained with the Bucher’s method also performs better compared to the Match. Comparing the original PWM built by the Bucher’s method with new mono-nucleotide PWM we can see a very little difference between the performances of the matrices. But if we compare performance of the initial PWM with those of the optimized di-nucleotide PWM we can see that the OF_r_ is much lower (i.e. specificity is much higher) for the di-nucleotide matrix with similar sensitivity. This can be observed from the Figure 7 where Match’s OF_r_ on the y-axis is around 0.007 with the sensitivity of ~60%. Yet OF_r_ for other PWMs at the Figure 7 is around 0.002 with ~60% of sensitivity. Even if we increase the sensitivity, the OF_r_ reaches up to around 0.004 even with 80% sensitivity which is far below from the OF_r_ of Match which is 0.007.

**Figure 8:**
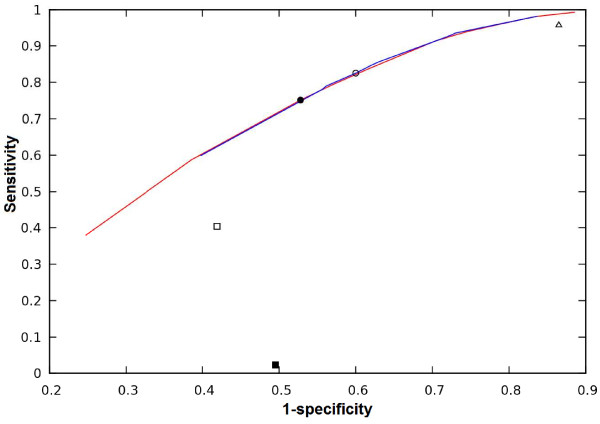
**A Receiver-Operator Characteristic curve (ROC) of the optimized PWM (mono-nucleotide and di-nucleotide) compared with the program Match.** The red and blue lines represent mono and di-nucleotide PWMs respectively. The filled and blank circles represent the optimized cutoff for mono and di-nucleotide PWMs. The empty triangle on the top right represents the Match minFN cutoff and the filled and the blank square represents the performance of Match minFP and minSUM cutoff.

We have also compared the performance of all the matrices considering specificity as proportion of true hits among all positive predictions using ROC curve (Figure 8). From the curve we can see that the performance of the new matrices is much better than those of Match with all the three matrix-specific cutoffs. The red and blue lines in Figure 8 represent the sensitivity and specificity of the new mono- and di-nucleotide PWMs respectively. The filled and blank circles represent the performance at the optimized cutoff for mono- and di-nucleotide PWMs respectively. The filled and the blank squares represent the performance of Match minFP and minSUM cutoffs respectively, and the blank triangle represents the performance of minFN cutoff. The superior performance of the optimized PWM is clearly seen from the Figure.

### Comparison of the new and the original PWM

The new mono-nucleotide matrix is similar to the original matrix with some insignificant differences for T in positions 1, 3, 6 (Table 1). The weight for nucleotide A is similar across all the positions. For nucleotide G there is no much difference except positions 3, 4 and 5. And for nucleotide C the differences are in positions 2, 5 and 6. The core consensus sequences (GATA) are similar for both new and original mono-nucleotide matrices.

**Table 1:** Comparison of original and new mono-nucleotide PWM

**A)**						
	**1**	**2**	**3**	**4**	**5**	**6**
A	25	0	61	0	39	15
T	20	0	1	58	19	8
G	4	62	1	5	4	37
C	14	1	0	0	1	3
A	0.40	0.0	0.97	0.0	0.62	0.24
T	0.32	0.0	0.02	0.92	0.30	0.17
G	0.06	0.98	0.02	0.08	0.06	0.59
C	0.22	0.02	0	0.0	0.02	0.05
A	0.00	-3.83	0.00	-4.12	0.00	-0.55
T	-0.021	-3.82	-4.10	0.00	-0.79	-1.16
G	-2.19	0.00	-4.47	-2.81	-2.63	0.00
C	-0.92	-4.11	-4.51	-4.47	-4.10	-2.50
	a/t	G	A	T	A	g/a
**B)**						
	1	2	3	4	5	6
A	27	0	66	0	43	16
T	22	0	1	63	20	8
G	4	67	1	5	4	41
C	15	1	0	0	1	3
A	0.40	0.0	0.97	0.0	0.63	0.24
T	0.32	0.0	0.01	0.93	0.29	0.12
G	0.06	0.99	0.01	0.07	0.07	0.62
C	0.22	0.01	0.0	0.0	0.01	0.04
A	0.00	-3.83	0.00	-4.13	0.00	-0.59
T	-0.19	-3.82	-3.48	0.00	-0.76	-1.27
G	-2.26	0.00	-3.84	-2.9	-2.73	0.00
C	-0.93	-3.49	-4.52	-4.48	-3.4	-2.61
	a/t	G	A	T	A	g/a

As was stated earlier in [18], the di-nucleotide PWM contains more information than mono-nucleotide matrix. We compared our new di-nucleotide PWM with the mono-nucleotide PWM (Table 2). For each di-nucleotide, the observed values are presented as first line and expected values are on the subsequent line of the Table 2. The expected values were calculated from the mono-nucleotide PWM. The observed frequencies were calculated from the binding sites used to build the new di-nucleotide PWM.

**Table 2:** Comparison of expected and observed di-nucleotide frequencies and di-nucleotide PWM

	**1**	**2**	**3**	**4**	**5**
AA	0	0	0	0	14
	0	0	0	0	11.75
AT	0	0	72	0	10
	0	0	71.04	0	5.88
AG	32	0	5	0	20
	30.91	0	5.64	0	30.12
AC	0	0	0	0	4
	0.46	0	0	0	2.2
TA	0	0	0	45	9
	0	0	0	46.28	5.47
TT	0	0	1	24	0
	0	0	1.08	21.53	2.73
TG	25	0	0	4	17
	25.18	0	0.09	4.31	14.01
TC	0	0	0	1	0
	0.38	0	0	1.08	1.03
GA	0	76	0	3	0
	0	75.55	0	3.67	1.09
GT	0	1	1	2	0
	0	1.14	1.08	1.71	0.55
GG	3	1	0	0	3
	4.58	1.14	0.09	0.34	2.8
GC	1	0	0	0	1
	0.07	0	0	0.09	0.21
CA	0	1	0	0	0
	0	1.13	0	0	0.27
CT	0	0	0	0	0
	0	0.02	0	0	0.14
CG	18	0	0	0	1
	17.17	0.02	0	0	0.7
CC	0	0	0	0	0
	0.26	0	0	0	0.05
AA	-4.62	-5.66	-6.16	-5.81	-0.16
AT	-4.14	-5.18	0.00	-5.33	-0.02
AG	0.00	-5.91	-3.39	-6.06	-0.05
AC	-4.41	-5.45	-5.94	-5.60	-1.20
TA	-4.02	-5.06	-5.55	0.00	0.00
TT	-4.58	-5.62	-4.72	-1.20	-4.17
TG	-0.09	-5.75	-6.24	-3.11	-0.06
TC	-4.69	-5.73	-6.22	-4.48	-4.27
GA	-4.69	0.00	-6.22	-3.38	-4.27
GT	-4.44	-4.08	-4.57	-3.54	-4.02
GG	-2.69	-4.83	-6.72	-6.38	-2.27
GC	-3.70	-6.14	-6.63	-6.29	-3.29
CA	-4.70	-4.34	-6.24	-5.89	-4.28
CT	-4.84	-5.89	-6.38	-6.04	-4.43
CG	-0.47	-5.80	-6.29	-5.95	-2.95
CC	-5.17	-6.22	-6.71	-6.37	-4.76
	AG/TG/CG	GA	AT	TA	TA/AT/AG/TG/AA

Optimized di**-**nucleotide frequency table and PWM. The observed frequencies are provided in the first line for each di-nucleotide, with the following line representing expected di-nucleotide frequencies (calculated from the mono-nucleotide frequencies). The presented are the frequencies of the di-nucleotides from the motifs selected from the interval -7 to 0.

If we compare the frequencies presented in the Table 2 we can see the variation in the last (5^th^) column. For example, the expected frequency for di-nucleotide AT at position 5 is 5.88 but the observed value (10) is much higher. The new di-nucleotide PWM includes more variations in the 1^st^, 5^th^ and 6^th^ positions.

GATA-3 is a factor from the family of DNA binding proteins GATA with consensus motif (A/T)GATA(A/G) [43-45]. From these results one can see that the core GATA motif is preserved by the two new matrices. In addition, the di-nucleotide PWM assigns the highest weight to the di-nucleotide AT and TA in the 5^th^ position, which results in the incorporation of nucleotide T in the 5^th^ and 6^th^ positions in the core consensus motif as compared to the mono-nucleotide PWM. However, like mono-nucleotide PWM, the di-nucleotide PWM also assigns higher weight to AA, TG and AG which confirms the presence of nucleotides A or G in the 6^th^ position. Like for the mono-nucleotide PWM, higher weights are assigned to CG, AG and TG, which confirms the variation of C, A and T at the 1^st^ position. The incorporation of C at the 1^st^ position, T at the 5^th^ position and T at the 6^th^ position makes the new consensus [A/C/T]GAT[A/T][A/T/G] which is different from those by the mono-nucleotide PWM ([A/T]GATA[G/A]).

### Genome-wide mapping of GATA-3 binding sites

The new PWMs can be used to search for novel putative binding sites [14,15]. We searched the human proximal promoters from EPD database to obtain novel GATA-3 binding sites with the new PWMs. The same promoter lengths were used as in the earlier sections. We applied the optimized threshold and scanned the promoter sequences of human genome finding a total of 4329 putative binding sites using di-nucleotide matrix and 7401 putative binding sites using mono-nucleotide matrix. Such a high number is not unexpected considering the short size of the motif. Some of these identified putative binding sites may be false positive and functionally irrelevant. To restrict the selection of non-functional binding sites we considered only the binding sites occurring in the optimized functional window (-7 to 0) of the promoter sequences (see above).

Table 3 shows the new sites found by our optimized di-nucleotide matrix. The functionality of some of these sites has been demonstrated in earlier publications. For example AGATTA and TGATAG have been cited in [28,29]. However they were missed by Match and are not included among GATA-3 binding sites in TRANSFAC and Jaspar. With minFN (minimized false-negative) Match could find some sites like TGATAG and GGATAT, but search with this cutoff is associated with a very low specificity. However with other cutoffs having higher specificity i.e. minFP (minimized false-positive) and minSUM (minimized sum of FP and FN) Match could not find any of the sites listed in the Table 3.

**Table 3:** Motifs discovered by di-nucleotide PWM from -7 to 0 [TSS]

**Motif**	**Score**	**Match**		
		**minFN**	**minSum**	**minFP**
TGATAG	-0.14	Found	Not found	Not found
AGATTA	-1.19	Not found	Not found	Not found
CGATTA	-1.66	Not found	Not found	Not found
GGATAT	-2.71	Found	Not found	Not found

New sites found in human promoter sequences by new PWMs and Match. The sites selected with highest score by the di-nucleotide PWM are shown. The column Motif shows the sites picked up from the human promoters in EPD database and the column Score shows the score given to these sites. The column Match shows the sites found by the Match program with the threshold provided in TRANSFAC as a minimum FN, minimum SUM and minimum FP level in the same database EPD.

Recently GATA-3 bound regions in the human genome in T-47D epithelial cell line derived from a mammary ductal carcinoma were submitted by the ENCODE Project Consortium [46] in the GEO expression database [47]. We have extracted the genomic regions from the BED file provided. To verify the performance of the new PWMs with the existing PWMs and with Match program we searched the PWMs in these bound regions of the genomic sequences. If the PWM could recognize any site in the extracted GATA-3 bound sequences then the number of these sequences is considered as the true positive. And the number of remaining sequences where PWM could not find any match is regarded as the level of false negatives. Thus we calculate the sensitivity. To examine the specificity we use sequences where GATA-3 is not bound as the negative dataset. To construct the latter we have extracted sequences of length 1000 bp from the same human genome reference (GRch37/hg19) from UCSC from where the GATA-3 bound sequences were extracted. The negative sequences were extracted so that they do not overlap with the bound sequences. We have mapped the PWM hits in these GATA-3-non-bound sequences and calculated the false positive rate using the number of hits in these sequences. The Table 4 shows the performance of each of the PWMs and the program Match. It is seen from the Table that sensitivity of the program Match with minFN is higher (96%) than for any other PWMs. However, its specificity is much lower. Our new di-nucleotide PWM has sensitivity of 83% with better specificity. The Table summarizes the total number of hits found by all the PWMs in both GATA-3 bound sequences and in not bound sequences. Since the GATA-3 bound sequences contain at least one GATA-3 binding site, we calculate the sensitivity as the proportion of sequences predicted as bound (i.e. bound sequences having any hits). The hits in the GATA-3 not bound sequences can be regarded as false predictions, yet we don’t know a total number of false positive predictions because some hits in the positive sequences may be also false. Hence it makes more sense to estimate the level of false positives by number of hits in the negative sequences than by usual specificity level (proportion of true hits among all positives) since the latter is hard to calculate. As one may see from the Table 4, total of 27158 sequences are found as GATA-3 bound sequences by the Match minFN which is 16% more than by the new di-nucleotide PWM and 27% more than by the new mono-nucleotide PWM. Nevertheless, the superiority of the new PWMs can be seen if we compare the PWMs in terms of the rate of false predictions from the GATA-3 not bound sequences. The match minFN gives 84% and 148% more false prediction than the new di-nucleotide PWM and new mono-nucleotide PWM respectively. In case of Match with more relaxed thresholds the sensitivity falls dramatically, while level of false positive hits in the non-bound sequences is comparable with those in the bound sequences.

**Table 4:** Performance of different PWMs in GATA-3 bound sequences and GATA-3 not bound sequences

**PWM**	**GATA-3 bound sequences**				**GATA-3 not bound sequences**		
	**Total hits**	**TP sequences**	**FN**	**Sensitivity**	**Total hits**	**Total sequences with hits**	**TN**
New-mono	45095	21289	7040	75%	39276	19760	8569
New-di	58409	23378	4951	83%	52897	22450	5879
Match_minFN	102257	27158	1171	96%	97461	27071	1258
Match_minFP	673	663	27666	2.38%	639	630	27699
Match_minSUM	14988	11436	16893	43%	12330	9846	18483

## Discussion

Since the prevalent positioning of the GATA-3 motif overlaps the TSS, it can be suggested that the GATA-3 motifs (GATA) and TSS-related motif share some bases. To check this possibility we have divided the promoters with respect to the Initiator element Inr (YYANWYY) with Promoter Classifier [39] and compared the z-score distribution of GATA-3 in both the Inr + and Inr- datasets. We found that in both the datasets there are some remarkable two peaks (one immediately upstream and another at -32 bp upstream of TSS) as mentioned earlier Additional file 1: Figure S2. Although the promoters having the Inr element have higher peak in the immediate upstream area compared to the promoters missing the Initiator, which may suggest some contribution of the Initiator to the higher peak in this area, the peak in the Inr-less promoters demonstrates an enrichment of genuine GATA-3 elements in that area.

The slope in the z-score distribution of the AT-rich factors like GATA-3, Ets-1 and TCF-1 is the manifestation of the under-representation of their binding sites around the functional window. If we plot the z-score distribution of GATA-3 further upstream i.e. up to 10 kb, the slope in the z-score starts approximately from 1 kb upstream of the TSS. The under-representation becomes more prominent closer to the functional window ( Additional file 1: Figure S1). The z-score distribution for the other transcription factors specific for T-cell (TCF-1 and Ets-1) also shows the similar pattern of distribution in the 10 kb promoter databases (data not shown).

Our new mono-nucleotide and di-nucleotide PWM were able to identify novel binding sites for GATA-3 factor ( Additional file 1: Table S1), and some of these sites found experimental confirmation in the previous literature. The novel sites found by the new PWM are not being found by Match and initial PWM with stringent cutoff. If we relax the cutoff we may get these novel sites found by the existing matrices, but then number of false positive predictions increases dramatically. The novel sites mentioned in our study were previously experimentally verified as the GATA-3 binding sites, and the mutations in the sites lead to the complete loss of their activity. These sites were not among the sites in the TRANSFAC database and hence were not a part of our training data set.

## Conclusions

The present work provides computationally refined PWMs for GATA-3 transcription factor along the lines established earlier [18]. Thus implementing the optimization method we can refine the existing TRANSFAC or Jaspar PWMs to new PWMs which outperform the existing PWMs. The PWMs thus obtained can be used to discover new binding sites. The optimized PWM is expected to help the researchers working with GATA-3 when we still have but a handful of experimentally confirmed binding sites. The present work also confirms the fact that the di-nucleotide PWMs provide viable alternative to the standard mono-nucleotide PWMs.

The high-throughput TFBS data is gradually revealed in the ChIP-chip and ChIP-Seq experiments. Yet the ChIP-chip method does not provide the data with high resolution necessary for building reliable PWM. The high throughput GATA-3 TFBS data is not published yet in any frequently used databases like TRANSFAC or Jaspar. To work with PWM for GATA-3 one still has to resort to the data from TRANSFAC and Jaspar, which are quite widely used as the best available datasets now despite known weaknesses. Therefore any scientist looking for a model to predict putative GATA-3 binding sites in sequences of interest is still limited by the available (even though somewhat inadequate) model to work with. This study focuses on the improvement of the existing GATA-3 PWM with the same limited resources. While we may someday be overwhelmed with binding site information for GATA-3 from technologies like ChIP-chip or ChIP-seq, at the present time our method provides substantially better alternative to the existing PWMs from TRANSFAC or Jaspar.

## Materials and methods

The method of optimization proposed in [18] used three starting input elements to build the new PWM, namely, an existing PWM, a database of promoter sequences and a set of experimentally verified TF sites. Consensus motifs can also be used instead of existing PWM and the considered database should be well populated with TFBS of interest. We have adopted this method with slight improvements.

### Building initial PWM

To build the initial PWM form the training set of experimentally defined binding sites, we used 63 experimentally defined motifs for human GATA-3 from Jaspar database [40,48,49]. The matrix information was adopted from [44]. As described in [16,18,50] to build the PWM we first find out the frequencies for each base at each position of the aligned known 63 GATA-3 binding sites. The frequencies are further converted into odds scores by dividing the observed frequency by expected frequency or the background frequency of each nucleotide at each position, averaged over the proximal promoters [50]. We have derived the expected frequencies using the formula described in [18]:

(1) $$e_{\mathrm{bi}}=\frac{\sum_{i=1}^{L}n_{\mathrm{bi}}}{L}$$

where *b* is one of 4 nucleotides (A,C,G or T) at position *i*, *n*_*bi*_ is the number of times base *b* occurs at the *i*^th^ position of the motif and *L* is the length of the sequence.

The expected frequencies were derived from the human promoter sequences from Eukaryotic Promoter Database (EPD) [31-33] release 105 (http://www.epd.isb-sib.ch/).

The database contains 1870 non-redundant experimentally verified human promoter sequences. We extracted 600 bp promoter sequences from this database, which comprise up to -499 positions upstream of the transcription start site (TSS) to position +100 downstream with TSS at 0. The promoters are aligned with respect to the TSS. Therefore the value for L in our case is 600, the length of the promoter area.

The positional distribution of the GATA-3 motif derived from the above database is also compared with Database of Transcription Start Sites (DBTSS) [34,35]. This database contains 32102 promoter sequences aligned with respect to the TSS. The rationale of using both databases (EPD and DBTSS) is that EPD is manually curated and is highly reliable, whereas DBTSS contains more promoters.

The weight for each position of the matrix is derived using the formula described in [18] which is a modification of Bucher’s formula:

(2) $$w_{\mathrm{bi}}=\ln\left(\frac{n_{\mathrm{bi}}}{e_{\mathrm{bi}}}+s_{i}\right)+c_{i}$$

Here *b* is one of the 4 nucleotides, *n*_*bi*_ is the number of times base *b* occurs at the *i*^*th*^ position of the motif, *c*_*i*_ is a constant providing column maximum value to be zero, *s*_*i*_ is a smoothing parameter preventing the logarithm of zero (or too small a value).

(The parameter *S*_*i*_ in Bucher’s formulae is used as the smoothing percentage.) We adopted the criteria as described in [18]: *S*_*i*_ = 0 if the first term under logarithm in Formula 2 is larger than $0.01\times \frac{n}{4\times e_{\mathrm{bi}}}$ and $s_{i}=0.01\times \frac{n}{4\times e_{\mathrm{bi}}}$ otherwise, where

(3) $$n=\sum_{b=1}^{4}n_{b}$$

To calculate weights for the di-nucleotide matrix we used the same Formula 2. In this case *b* represents one of the 16 di-nucleotides, *n*_*bi*_ is the number of times di-nucleotide *b* is present in position *i* of the motif and *e*_*bi*_ is the expected frequency of the di-nucleotide *b* at the *i*^*th*^ position, *c*_*i*_ and *s*_*i*_ have the same meaning as for the mono-nucleotide PWM, *s*_*i*_ = 0 if the first term under logarithm in Formula 2 is larger than$0.01\times \frac{n}{16\times e_{\mathrm{bi}}}$ and $s_{i}=0.01\times \frac{n}{16\times e_{\mathrm{bi}}}$ otherwise, where

(4) $$n=\sum_{b=1}^{16}n_{b}$$

The mono-nucleotide matrix thus built has 4 rows where each row represents each nucleotide and the columns represent positions inside the motif. The di-nucleotide matrix has 16 rows, with each row representing each di-nucleotide. The number of columns of the matrix represents the length of the motifs which is less by one for di-nucleotide PWM comparing to those for mono-nucleotide.

To calculate the weight score *S* for a specific sequence we use the formula:

(5) $$S=\sum_{i=1}^{L_{m}}w_{\mathrm{bi}}$$

where *L*_*m*_ is the length of PWM, *w*_*bi*_ is the weight of nucleotide *b* at position *i* in the PWM. For di-nucleotide matrix we use the same formula with *L*_*m*_ *= L*_*m*_ -1 instead of *L*_*m*_ and *w*_*bi*_ represents weight of di-nucleotide.

### Finding the functional window and optimization of the matrix

To obtain the positional distribution of the GATA-3 motif we compare the observed occurrence frequency of the GATA-3 motif with its background or expected frequency along the promoter sequences. The background frequency is determined by shuffling each sequence from the promoter database which results in a randomized DNA sequence with the same nucleotide content.

Shuffling of the sequences was done by cutting each sequence in randomly chosen positions into randomly chosen smaller fragments and rearranging these fragments. The sequences were fragmented with segment lengths from 1 bp to 10 bp. This step was repeated 100 times and the whole process was repeated 100 times. The EPD database was used for the shuffling; therefore the shuffled sequence database contained sequences of same number and length. Since we exclusively considered the promoter region for shuffling, we thereby preserved the proportion of all the nucleotides in the shuffled sequences as that in the promoter datasets. The reason to preserve the proportion is to retain the GC-rich property of the promoter in the shuffled sequences. The GC-proportion was checked with the help of program called “geecee” from the collection of program suite EMBOSS [51] after shuffling the promoter sequences and found to be similar. After shuffling the sequences to compare the proportion of CpG di-nucleotide for first-order dependencies we have used the Promoter Classifier [39] to see how many sequences in both the original and the shuffled promoter sequences are CpG-rich (contain CpG islands). We found that both the datasets have similar number of CpG rich sequences. Moreover, we have further examined the shuffled sequences to see if we reduced the number of CpG while shuffling the sequences. To do this we have used the “cgpreport” program from EMBOSS [51] and calculated the average CpG per sequences in both shuffled and original promoter sequences. From this we found that the average number of CpG in the shuffled sequences is consistent with those in the original promoters.

To identify the area where the GATA-3 binding site motif is over-represented along the aligned promoter sequences, we looked into the distribution of the z-score derived as

(6) $$z-score=\frac{\left(Obs-Exp\right)}{\sqrt{Exp}}$$

where *Obs* is the observed occurrence frequency of GATA-3 element in the promoter sequences and *Exp* is the expected occurrence frequency as found in the shuffled promoter sequences.

The occurrence frequencies were calculated as $OF_{i}=\frac{n_{i}}{N_{s}}$ where *n*_*i*_ is the number of promoters containing considered motif starting at position *i* and *N*_*s*_ is the number of sequences.

The area where the occurrence of the GATA-3 motif is statistically higher than expected, which is represented by z-scores ~3 or higher, is regarded as the initial “functional window” (Figure 1). This region shows the occurrence frequency of GATA-3 binding sites much higher than the background or expected frequency.

We assume that statistically significant occurrence of the sites in the “functional window” reflects importance of this window in biological function. The functional window thus obtained is the initial approximate interval from where the new sites can be incorporated to build a new PWM. The final matrix after optimization would define the exact functional window.

### Calculation of a new GATA-3 PWM from the existing PWM

PWMs are routinely used for prediction of the binding affinities for TFs to a segment of DNA sequence in prokaryotes and eukaryotes [52-56]. TRANSFAC database holds numerous PWMs for large variety of TFs. However the majority of the existing PWMs provide a low level of both sensitivity and specificity [55]. Therefore the need to optimize PWM parameters in order to improve its performance is essential. The method developed by Gershenzon et al. [18] iteratively modifies the PWM by incorporating new putative binding sites located inside the identified functional window from the promoter sequence database until the best possible correlation coefficient is achieved as described below. We follow this method in the present study.

We start with the initial PWM built from the 63 GATA-3 experimentally defined motifs adopted from Jaspar, as described in the previous section. As a control set of sites, we use the 26 unique motifs from the 63 experimentally defined GATA-3 binding sites in human genes from Jaspar. The removal of redundancy from the experimental motifs was important to avoid any biasness toward any motif. The method also utilizes a database to incorporate new putative binding sites of interest to build new PWM. We have used the EPD for this purpose.

Since the initial PWM is not provided with a given cutoff we determine the cutoff value from the correlation coefficient (CC) distribution. The CC is calculated as:

(7) $$CC=\frac{\left(TP\times TN\right)-\left(FN\times FP\right)}{\sqrt{\left(TP+FN\right)\times \left(TN+FP\right)\times \left(TP+FP\right)\times \left(TN+FN\right)}}$$

CC is calculated for each cutoff starting from a very stringent threshold and relaxing the threshold until we get the maximal CC. To calculate CC here, we designate TP as the number of sites from the experimentally defined dataset positively identified by the matrix with a given cutoff. FN is defined as the difference between the total number of sites in the experimental dataset and TP. We designate negative sites as all possible sites from the shuffled sequence datasets, which can be calculated as 594 × 1870 where 594 is length of the shuffled promoter sequence (600) minus the length of the matrix and 1870 is the number of sequences in the shuffled dataset. We define FP as the sites picked up as positive from the total negative sites and TN as the difference between the total negative sites and FP.

The method starts with extracting putative binding sites for GATA-3 based on existing PWM with the cutoff determined at the above step. The PWM extracts putative binding sites from inside the identified initial functional window. The functional window was defined comparing the occurrence frequency distribution of the GATA-3 binding sites against the shuffled sequences. A new matrix is built from these aligned sites using the formulae described in [18]. These sites do not need any additional alignment as they are of similar length. The new matrix is obtained from new sites extracted by the original matrix with the given cutoff and sensitivity, from the functional window.

Sensitivity was calculated as:

(8) $$Sensitivity=\frac{TP}{\left(TP+FN\right)}$$

The optimization is done in three levels, as follows: cutoff value, then motif length and finally functional window.

The objective function that is optimized in this method is the correlation coefficient (Formula 7). This criterion utilizes all the four parameters: true positives, false positives, true negatives and false negatives.

The definitions of the TP, FP, TN and FN for the optimization procedure are slightly different from the previously described.

The TP here is defined as the number of sites positively identified by the new matrix from the given functional window identical to the sites extracted by the original matrix. FP is defined as the difference between the total number of sites identified as positive by the new matrix and the number of sites identified as positive by the original matrix. FN is defined as the difference between the total number of sites from the experimental dataset recognized by the original matrix as positive and the TP. And TN is the total number of possible sites from the functional window subtracting TP, FP and FN:

(9) $$FP=N_{\mathrm{new}}-TP\text{,}$$

(10) $$FN=N_{\mathrm{Original}}-TP\text{,}$$

(11) $$TN=l_{w}\times N_{s}-TP\_FP-FN$$

CC is calculated every time after building a new matrix by changing any parameters. (See the flow chart of the adopted optimization process in [18] [Figure 2 for details.)

First the optimal cutoff value is obtained for the given position and size of the functional window and for the given motif length. This is attained by calculating CC parameter for every changed cutoff value. The cutoff value varies around the initial given cutoff value. The range we have used is from -0.5 to -4.0 with the increment of 0.1. The cutoff value is considered to be optimal where the CC reaches the maximum. Next, the length of the matrix is varied while the optimal cutoff value is kept. If the CC reaches higher value than at the previous step, the modified length is considered as optimal at the current stage. Thus we obtain a modified matrix with optimal modified length and cutoff values. This modified matrix is regarded as the initial matrix for further process of optimization. The optimization cycle continues with this new initial matrix and all the aforementioned steps are repeated. This continues until we reach the maximal CC = 1. Usually it takes 6 to 12 cycles for the matrix to converge, which is consistent with the previous work [18]. After that we proceed to a new functional window by increasing or decreasing its length by 1 bp from either side. The CC is maximized again for each length and position of the functional window. This process is repeated for each considered functional window. Finally we would get a PWM with optimized cutoff value and matrix length from optimal functional window. From this pool of new matrices we use the criteria of sensitivity and specificity to select the best.

The parameters TP, TN, FP and FN described earlier are internal parameters of the procedure, and they are not used to evaluate the sensitivity and specificity of the final PWM. The sensitivity of the optimized PWM is calculated as the number of experimentally confirmed sites recognized by the new matrix. And to calculate the specificity we use the occurrence frequencies of predicted TFBS in the randomized sequences. We assume that the sites recognized as positive from the randomized sequences are the false positives. We calculate occurrence frequency as the average number of positive predictions per bp in the random shuffled dataset:

(12) OF=∑TotalpredictionNL

where *N* is the total number of sequences in the shuffled sequences database and *L* is the length of the sequence subtracting the length of PWM. We will use the notation *OF*_*r*_ to designate occurrence frequency calculated from shuffled sequence dataset. Therefore higher the occurrence frequencies from the shuffled sequences are, lower is the specificity. Now we choose the matrix resulted from the process of optimization that has sensitivity and specificity higher than the initial PWM.

## Abbreviations

PWM: Position weight matrix; TFBS: Transcription factor binding site; CC: Correlation coefficient; TP: True positive; TN: True negative; FP: False positive; FN: False negative; EPD: Eukaryotic Promoter Database; DBTSS: Database of Transcription Start Sites; ROC: Receiver-Operator Characteristic; OF: Occurrence frequency; OF_r_: Occurrence frequency from randomized sequences; ENCODE: Encyclopedia of DNA Elements; TCR: T cell receptor; Th: T helper cell; GEO: Gene Expression Omnibus.

## Competing of interests

The authors declare that they have no competing interests.

## Authors' contributions

SN. performed calculations, analyzed the results and wrote the manuscript. II. conceived the study and directed the work, including data analysis, figure assembly and manuscript writing. Both authors read and approved the final manuscript.

## Supplementary Material

Additional file 1 **The following additional data are available with the online version of this paper (all included in one file).** Additional file 1: Figure S1: z-score distribution of GATA-3 across 10 kb upstream of the EPD promoters. Additional file 1: Figure S2: Comparison of the GATA-3 z-score distribution in promoters divided with respect to the presence of Initiator element. Additional file 1: Table S1: The novel putative GATA-3 sites discovered from the EPD with location in the promoter sequences. Click here for file
