# Supplementary material for: Optimizing the GATA-3 position weight matrix to improve the identification of novel binding sites
Source: BMC Genomics. 2012 Aug 22;13:416. doi: 10.1186/1471-2164-13-416 (PMC3481455; doi:10.1186/1471-2164-13-416)
Supplement: Additional file 1 — The following additional data are available with the online version of this paper (all included in one file). Additional file 1: Figure S1: z-score distribution of GATA-3 across 10 kb upstream of the EPD promoters. Additional file 1: Figure S2: Comparison of the GATA-3 z-score distribution in promoters divided with respect to the presence of Initiator element. Additional file 1: Table S1: The novel putative GATA-3 sites discovered from the EPD with location in the promoter sequences. [file 1471-2164-13-416-S1.pdf]

Additional Figure 1: z-score distribution of GATA-3 across 10kb upstream.

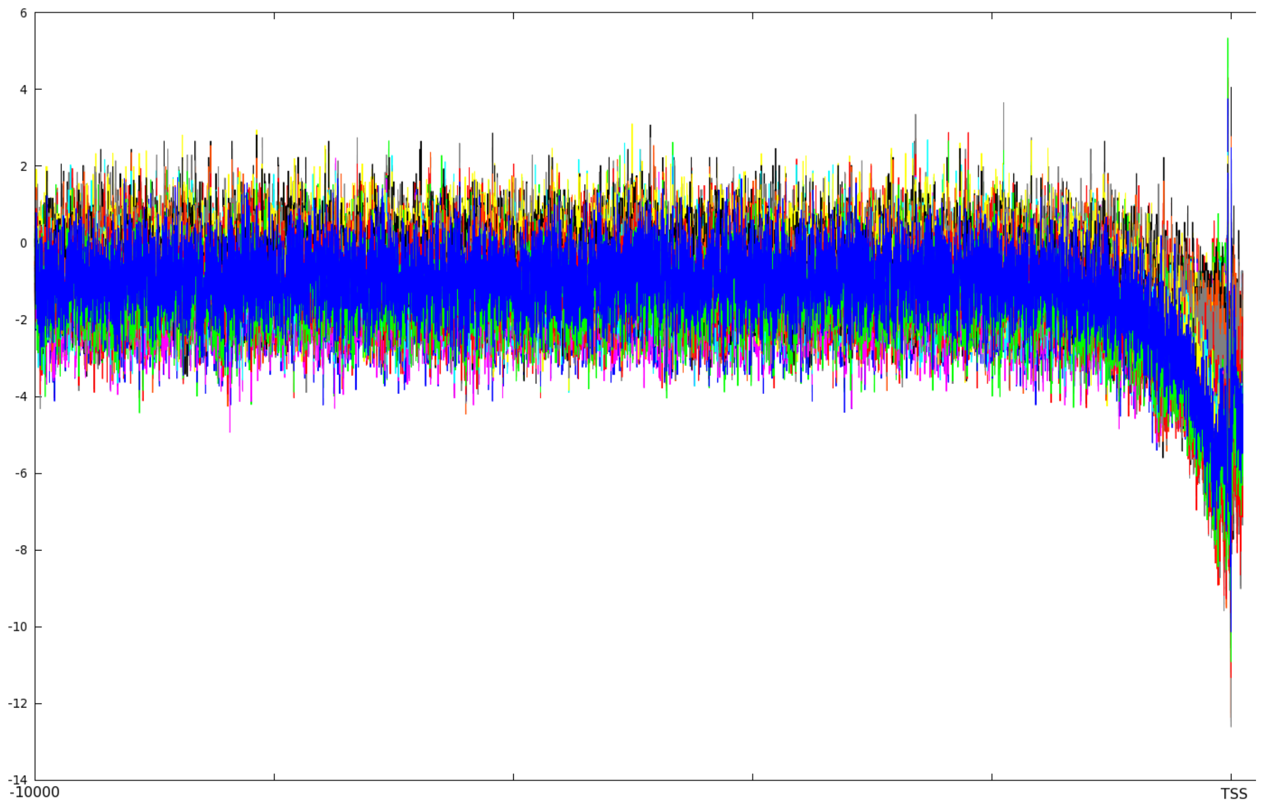

z-score distribution of GATA-3 across 10kb upstream of the EPD promoters. The slope starts around 1kb upstream of the TSS. Different colours represent different threshold starting from stringent to relaxed.

Additional Figure 2: Comparison of the GATA-3 z-score distribution in promoters divided with respect to the presence of Initiator element.

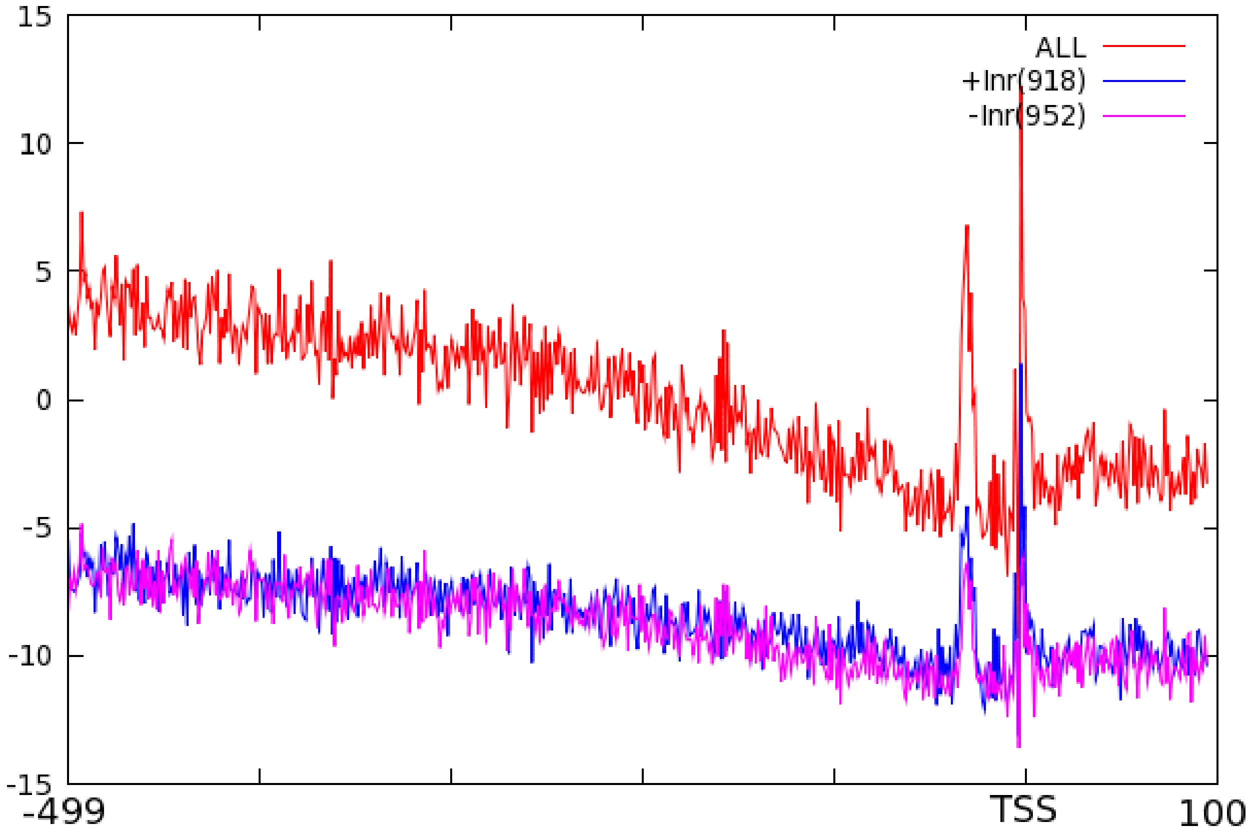

z-score distribution of GATA-3 transcription factor across the divided promoter sequences from EPD depending on presence of Initiator element. The x-axis shows the length of the promoter (-499 to +100). Magenta plot is the distribution of GATA-3 z-scores in the promoters without Initiator element (952), blue plot is the distribution of GATA-3 z-scores in the promoters having Initiator element (918) and the red plot is the distribution in all the 1870 promoters.

Additional Table 1: The novel putative GATA-3 sites discovered from the EPD with location in the promoter.

EPD_id	Site	Location	Strand	Score	Gene_name	Product
EP37014	CGATAA	-7	(+)	-0.631690	HS_RPS15	Ribosomal protein S15 [rat insulinoma gene].
EP15034	GGATAT	-5	(+)	-2.709070	HS_MMP1	Collagenase, Matrix metalloproteinase-1, MMP1 or CLG gene.
EP70008	AGATTA	-6	(+)	-1.192390	HS_ACP2	Lysosomal acid phosphatase 2.
EP40002	GGATAT	-1	(-)	-2.709070	HS_C5	Complement, C5 gene.
EP73020	AGATTG	-1	(+)	-1.250360	HS_TOSO	Regulator of Fas-induced apoptosis.
EP73066	GGATAT	-1	(-)	-2.709070	HS_MMS19L	MMS19-like (MET18 homolog, S. cerevisiae).
EP73436	TGATAG	-3	(-)	-0.142640	HS_MFAP1	Microfibrillar-associated protein 1.
EP73608	GGATAT	-5	(-)	-2.709070	HS_AHCY	S-adenosylhomocysteine hydrolase.
EP73694	AGATTA	-2	(-)	-1.192390	HS_NASP	Nuclear autoantigenic sperm protein (histone-binding) , transcriptvariant 2.
EP73895	GGATAT	-5	(+)	-2.709070	HS_RNF13	Ring finger protein 13.
EP73987	CGATTA	0	(+)	-1.660520	HS_MLANA	Melan-A.
EP74587	AGATTA	-6	(+)	-1.192390	HS_TTID	Titin immunoglobulin domain protein (myotilin).

The sites discovered from the EPD with location in the promoter sequences.
